# Mitochondrial Haplogroups and Risk of Pulmonary Arterial Hypertension

**DOI:** 10.1371/journal.pone.0156042

**Published:** 2016-05-25

**Authors:** Samar Farha, Bo Hu, Suzy Comhair, Joe Zein, Raed Dweik, Serpil C. Erzurum, Micheala A. Aldred

**Affiliations:** 1 Lerner Research Institute, Cleveland Clinic, 9500 Euclid Avenue, Cleveland, Ohio, United States of America; 2 Respiratory Institute, Cleveland Clinic, 9500 Euclid Avenue, Cleveland, Ohio, United States of America; 3 Genomic Medicine Institute, Cleveland Clinic, 9500 Euclid Avenue, Cleveland, Ohio, United States of America; Vanderbilt University Medical Center, UNITED STATES

## Abstract

Pulmonary arterial hypertension (PAH) is a serious and often fatal disease. It is a panvasculopathy of the pulmonary microcirculation characterized by vasoconstriction and arterial obstruction due to vascular proliferation and remodeling and ultimately right ventricular failure. Mitochondrial dysfunction is a universal finding in pulmonary vascular cells of patients with PAH, and is mechanistically linked to disease origins in animal models of pulmonary hypertension. Mitochondria have their own circular DNA (mtDNA), which can be subgrouped into polymorphic haplogroup variants, some of which have been identified as at-risk or protective from cardiovascular and/or neurodegenerative diseases. Here, we hypothesized that mitochondrial haplogroups may be associated with PAH. To test this, mitochondrial haplogroups were determined in a cohort of PAH patients and controls [N = 204 Caucasians (125 PAH and 79 controls) and N = 46 African Americans (13 PAH and 33 controls)]. Haplogroup L was associated with a lower rate of PAH as compared to macrohaplogroups N and M. When haplogroups were nested based on ancestral inheritance and controlled for age, gender and race, haplogroups M and HV, JT and UK of the N macro-haplogroup had significantly higher rates of PAH compared to the ancestral L (L0/1/2 and L3) (all p ≤ 0.05). Overall, the findings suggest that mitochondrial haplogroups influence risk of PAH and that a vulnerability to PAH may have emerged under the selective enrichment of specific haplogroups that occurred with the migration of populations out of Africa.

## Introduction

Genetic studies and fossil evidence support the hypothesis of a single origin to modern humans [1–4]. The evolutionary development of maternally inherited mitochondrial DNA (mtDNA) supports the hypothesis through the identification of a common maternal ancestor who lived in sub-saharan Africa 200,000 years ago. Over subsequent generations, her daughters accumulated mtDNA mutations, which produced characteristic clusters of variants within maternal lineages known as mitochondrial haplogroups. As humans migrated out of Africa, it is hypothesized that variants beneficial in the different and colder climates with better survival and/or reproduction in populations led to the current enrichment of specific haplogroups in geographic locations around the world [4–8]. The L haplogroup found in Africans is the primary and most ancient haplogroup. The N haplogroup population, derived from L, is found in Europe and harbors variants associated with lesser mitochondrial coupling efficiency and greater heat production [5, 9, 10]. It is speculated that N and the branches derived from it, became enriched for heat-production variants under selective pressure with migration to colder climate of Europe [5, 7, 10].

In general mtDNA polymorphic haplogroup variants are nonpathological, but some are identified as at-risk or protective related to bioenergetic and metabolic functions [8]. For example, haplogroup N has been associated with invasive breast cancer, whereas haplogroup U with prostate and renal cancers [11, 12]. In this context, metabolic derangements in the pulmonary vasculature and the right ventricle are universal findings in pulmonary arterial hypertension (PAH) [13–16]. PAH is a serious and often fatal disease. It is a panvasculopathy of the pulmonary microcirculation characterized by vasoconstriction and arterial obstruction due to vascular proliferation and remodeling and ultimately right ventricular failure [17]. Studies identify a metabolic shift to aerobic glycolysis in the lungs and hearts of PAH patients [15, 18]. The metabolic changes are associated with increased hypoxia inducible factors (HIF)-1α expression in pulmonary vasculature and cardiomyocytes in PAH [13, 19]. Mitochondrial function regulates the HIF response, and mitochondrial abnormalities are present in PAH, *e*.*g*. lower than normal cellular respiration, hyperpolarized mitochondrial membrane potential, decreased expression of electron transport chain complex I and mitochondrial superoxide dismutase [13, 14, 16, 19–21]. Several case reports have linked syndromic childhood-onset pulmonary hypertension with pathogenic mutations in mitochondrial genes or nuclear-encoded mitochondrial proteins [22–29]. However, the possibility that common mitochondrial variants may modulate risk for PAH has not been investigated. Based on the known alterations in mitochondrial function in PAH and the metabolic abnormalities in PAH, we hypothesized that mitochondrial haplogroups that influence cellular bioenergetics may carry vulnerability for the development of PAH.

## Materials and Methods

This study was approved by the Cleveland Clinic Institutional Review Board and all subjects provided written informed consent. DNA samples collected from patients with PAH and healthy controls for previous studies for which subjects consented for genetic testing were analyzed. Only Caucasians and African-Americans were included. Nine Asian subjects with PAH were excluded because there were only 2 Asians in the control group. Thirteen individuals of Hispanic ethnicity (4 controls and 9 patients) were excluded because they did not self-report their race. Where multiple samples were available from related individuals with the same maternal ancestry, a single sample was randomly selected for analysis to ensure that each mitochondrial lineage was included only once. Paternal relatives were included. DNA was extracted from blood samples using the Qiagen DNA blood mini kit (Qiagen, Valencia, CA) or from saliva using the Oragene kit (DNA Genotek, Ottawa, ON, Canada). The single nucleotide polymorphisms (SNP) used to define each haplogroup are listed in Table 1. mtDNA SNPs defining the different haplogroups were genotyped using realtime PCR using allele-specific hydrolysis probes and Taqman PCR mastermix (Life Technologies) on all samples. Primer and probe sequences were obtained from Benn *et al* [30]. PCRs were set-up robotically using an Eppendorf EpMotion liquid handling station and genotyped in 384-well plates on a Roche Lightcycler 480 instrument. Genotype calling is performed using the manufacturer’s software. The A12308G SNP (haplogroups U and K) was genotyped by PCR amplification using the primers GCTCACTCACCCACCACAT (forward) and GGATGCGACAATGGATTTTA (reverse), and then sequenced on an Applied Biosystems 3730xl capillary sequencer. Mitochondrial haplogroups were assigned on the basis of consensus tagging SNPs as established in the mitochondrial haplogroup literature and summarized on the Mitoweb website. For the samples that were not assigned to a haplogroup by these assays (n = 66), we performed long-range PCR amplification of the whole mitochondrial genome in two overlapping fragments, as previously described [31] and sequenced on the 3730xl [32, 33]. The long-range PCR amplification allowed further haplogroup assignment based mainly on subclasses of the L macrohaplogroup. Analysis for mutations in *BMPR2*, *CAV1*, *KCNK3* and *EIF2AK4* was performed by Sanger sequencing.

**Table 1:** Single nucleotide polymorphisms used to define each haplogroup.

	Base Position													
Mitochondrial Haplogroups	663	1719	3594	4580	7028	8251	9055	10398	10400	12308	12612	15607	16391	other
A	G	G	C	G	T	G	G	A	C	A	A	A	G	
H	A	G	C	G	C	G	G	A	C	A	A	A	G	
I	A	G	C	G	T	G	G	A	C	A	A	A	A	
J	A	G	C	G	T	G	G	G	C	A	G	A	G	
K	A	G	C	G	T	G	A	G	C	G	A	A	G	
L0/L1/L2	A	G	T	G	T	G	G	G	C	A	A	A	G	
L3	A	G	C	G	T	G	G	G	C	A	A	A	G	A10819 or A10086G
M	A	G	C	G	T	G	G	G	T	A	A	A	G	
T	A	G	C	G	T	G	G	A	C	A	A	G	G	
U	A	G	C	G	T	G	G	A	C	G	A	A	G	
V	A	G	C	A	T	G	G	A	C	A	A	A	G	
W	A	G	C	G	T	A	G	A	C	A	A	A	G	
X	A	A	C	G	T	G	G	A	C	A	A	A	G	

### Statistical Analysis

The distribution of the mitochondrial haplogroups was summarized as percentages and frequencies by race and disease status. The rates of PAH were compared between race groups using the Chi-square test. The study has a power of 80% to detect a 22.5% difference, assuming a 61.3% PAH rate for whites (two sided alpha = 0.05). The rates of PAH were estimated for individual mitochondrial haplogroup and the Chi-square test was used to compare the rates from different haplogroups. To test the protective effect of the L (L0/L1/L2 and L3) haplogroup within African Americans only, the study (13 PAH and 33 control individuals) has a power of 80% to detect a difference of 28% in the representative rate of haplogroup L for controls, assuming that the representative rate is 69% for PAH individuals.

To further adjust the comparison results for patient characteristics, a logistic regression model with the disease status (PAH or control) as the outcome was then applied, where the covariates included the haplogroups, age, gender and race. In this model, the original haplogroups were regrouped into L (L0/1/2 and L3), HV, M, UK, JT and AIWX according to the branching points and the temporal evolution. Odds ratios were computed with the L group as the reference. All analyses were conducted using R-studio (Boston, MA). Statistical significance was established with a two-sided p-value < 0.05.

## Results

### Study Population

Deidentified DNA samples from PAH patients and healthy controls at the Cleveland Clinic were analyzed. All subjects signed Cleveland Clinic IRB-approved consent forms for genetic testing. Of the 262 samples, 95% (N = 250) were assigned to specific mitochondrial haplogroups; 5% of samples were not classifiable, or could not be genotyped due to poor quality DNA, and were excluded. The samples included for analyses (N = 250) were representative of the racial distribution mainly in Northern Ohio and included 204 Caucasians (125 PAH and 79 controls) and 46 African Americans (13 PAH and 33 controls). Healthy controls did not have history of lung disease and were not on medications. PAH was confirmed by documentation of pulmonary hypertension by right heart catheterization in 65% of individuals, or estimated by echocardiogram in 35%. All PAH participants were Group 1 or 1’ (67 Idiopathic, 21 Heritable, 41 Associated PAH, 3 pulmonary veno-occlusive disease, 6 non-subtyped PAH). Three African American and 16 Caucasian patients, including 1 with pulmonary veno-occlusive disease and 1 with pulmonary capillary hemangiomatosis, had mutations in the bone morphogenetic protein receptor type 2 (BMPR2), Caveolin 1 (CAV1) or EIF2AK4 gene. PAH participants were generally older than controls [Age (years ± SD): PAH 47± 15, Controls 37 ± 14; p < 0.001]. As expected, the PAH sample was predominantly female (72%); and control sample was ~50% of each sex [Female/Male: PAH 100/38, Controls 60/52; Chi-square p = 0.002].

### Mitochondrial Haplogroups in PAH

As previously reported, the most frequent haplogroup among Caucasians was H (44%) and among African-descent Americans L (L0/L1/L2) (65%). Self-reported race was not uniformly concordant with mitochondrial assignment of maternal ancestry. Although L haplogroups were predominant in 89% of African Americans, M subtypes were found in 2% and N subtypes in 9%. This is consistent to what has been reported in the literature. An earlier study showed that 86% of African Americans were of the L haplogroup whereas about 3% belong to the M haplogroup and 11% to the N haplogroup [34]. N subtypes were predominant in 96% of Caucasians, but L was found in 1% and M haplogroups in 3%. Although our overall sample was representative of the local population, there was a significant difference in racial distribution within PAH and controls with less African Americans in the PAH group as compared to the control group [Caucasian/African American: PAH 125/13, Controls 79/33; Chi-square p < 0.001]. Thus it was not surprising that there was a significant difference in haplogroup distribution between controls and PAH (Chi-square p = 0.0035). Gender was not related to haplogroup distribution (p > 0.1).

**Fig 1:**
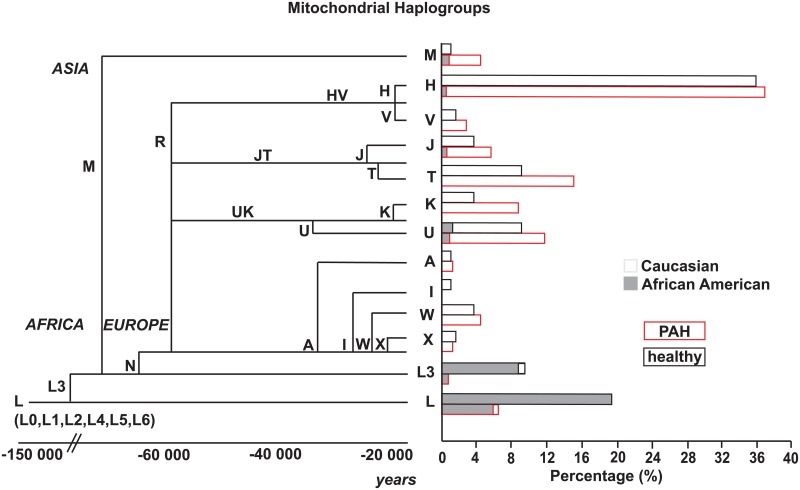
Mitochondrial haplogroup tree with the Eve mitochondrial haplogroup L originating in Africa and migrating out as haplogroups M and N acquired variants allowing for adaptation to different climates and geographical conditions. The different haplogroups are shown according to the timeline and their branching from the major macrohaplogroups M and N. The graph shows percentage of PAH or control populations by haplogroup distribution.

Since PAH and control groups had different proportions of Caucasians and African Americans, each self-reported racial group was analyzed separately [Table 2, Fig 1]. Strikingly, haplogroup L (L0/L1/L2 and L3) was significantly more represented among African American healthy controls (97%) as compared to African American individuals with PAH (69%) [OR = 14.2, CI = 1.4–143.7; p = 0.02]. This indicates a possible protective role of the L haplogroup, or an at-risk effect of non-L haplogroups. No significant association was found between PAH and haplogroup within the Caucasian group (all p > 0.1).

**Table 2:** Distribution of mitochondrial haplogroups by race and disease.

	Caucasian (N = 204)		African American (N = 46)	
Mitochondrial Haplogroups	PAH (N = 125)	Controls (N = 79)	PAH (N = 13)	Controls (N = 33)
**A**	2 (1.6%)	1 (1.27%)	0 (0%)	0 (0%)
**H**	50 (40%)	40 (50.63%)	1 (7.69%)	0 (0%)
**I**	0 (0%)	1 (1.27%)	0 (0%)	0 (0%)
**J**	7 (5.6%)	4 (5.06%)	1 (7.69%)	0 (0%)
**K**	12 (9.6%)	4 (5.06%)	0 (0%)	0 (0%)
**L0/L1/L2**	1 (0.8%)	0 (0%)	8 (61.54%)	22 (66.67%)
**L3**	0 (0%)	1 (1.27%)	1 (7.69%)	10 (30.30%)
**M**	5 (4%)	1 (1.27%)	1 (7.69%)	0 (0%)
**T**	21 (16.8%)	10 (12.66%)	0 (0%)	0 (0%)
**U**	15 (12%)	9 (11.39%)	1 (7.69%)	1 (3.03%)
**V**	4 (3.2%)	2 (2.53%)	0 (0%)	0 (0%)
**W**	6 (4.8%)	4 (5.06%)	0 (0%)	0 (0%)
**X**	2 (1.60%)	2 (2.53%)	0 (0%)	0 (0%)

### Mitochondrial Haplogroups and Ancestry in PAH

Analyses of haplogroups were initially performed with the assumption of independent effects comparing each haplogroup to the remaining pooled haplogroups. However, the mitochondrial haplogroup tree with the Eve mitochondrial haplogroup L originating in Africa and migrating out as haplogroups M and N is well defined: founding L-haplogroups gave rise to N and M, which subsequently gave rise to other discrete branches at different time points [Fig 1]. This allowed comparison of haplogroups to the ancestral L0/L1/L2. In this analysis, haplogroups H, U, J, T, K and M had higher rates of PAH than the ancestral L (all p < 0.05). When this analysis was performed controlling for race, age and sex, haplogroup M continued to have a significantly higher rate of PAH as compared to L0/L1/L2 [OR = 17.4, CI = 1.1–272.6; p = 0.04]. To further assess whether there was loss or gain of a specific variant at a specific branching time point, major-haplogroups or clusters were evaluated within collective branches based on their branching points and temporal evolution, as: L (L0/1/2 and L3), M, HV, JT, UK and AIWX (others). Overall, haplogroup clusters were different among PAH and controls (Chi-square p < 0.001). The major-haplogroups did not differ between PAH and controls within Caucasians (p = 0.5); however there was a trend within African American (p = 0.06). After adjusting for age, race and sex, haplogroup branches M, JT, HV and UK had significantly higher rates of PAH compared to L (L0/1/2 and L3) (all p < 0.05)[Table 3]. We further analyzed difference within haplogroup clusters in comparison to the major cluster in each individual race. For African Americans, the analysis was limited because of the small numbers. Within Caucasians, there was no significant difference among different haplogroup clusters when compared to the most frequent haplogroup H/HV (all p> 0.05) [Table 4].

**Table 3:** Odds ratio (OR) of having PAH in comparison to haplogroup L (L0/1/2 and L3) after controlling for gender, age, and race. Haplogroups were grouped based on their branching points and temporal evolution into M, HV, JT, UK and the others (AIWX).

Mitochondrial Haplogroups	OR	95% C.I.	p-value
**HV**	10.72	1.2–95.78	0.034
**M**	47.93	2.88–797.01	0.007
**UK**	15.59	1.97–123.12	0.009
**JT**	10.81	1.4–83.39	0.022
**AIWX**	0.33	0–2.38E+187	0.996

**Table 4:** Odds ratio (OR) of having PAH in comparison to haplogroup HV after controlling for gender and age within Caucasians.

Mitochondrial Haplogroups	OR	95% C.I.	p-value
**L/L3**	0.57	0-Inf	1
**M**	3.41	0.27–43.15	0.343
**UK**	1.52	0.42–5.47	0.523
**JT**	0.93	0.27–3.23	0.906
**AIWX**	1.06	0-Inf	1

### Mitochondrial haplogroups independent of genetic mutations predisposing to PAH

A mutation in *BMPR2* or another heritable PAH gene substantially increases the risk of developing PAH, from an incidence of about 1 per million of the population for idiopathic PAH to about 27% in gene carriers [35]. To eliminate any potential bias or masking effect of these mutations, we excluded individuals with a known family history and/or identified mutation and then re-analyzed the remaining samples (N = 229). After adjusting for age, race and sex, haplogroup M continued to have higher rates of PAH compared to L0/1/2 [OR = 19.3, CI = 1.15–322.8; p = 0.04]. Similarly upon adjusting for age, race and sex, comparison of M, HV, JT, UK and AIWX to the founder L continued to show that M, JT and UK had significantly greater numbers of PAH (all p ≤ 0.05) with a trend for HV to have increased rates of PAH (p = 0.08).

## Discussion

This study identifies an association of PAH with mitochondrial haplogroup M and clusters UK, HV and JT compared to the founder haplogroup L. Based upon mtDNA evolution, the findings indicate that populations may have acquired a vulnerability to PAH as the ancestral L haplogroup gave rise to M and N. It is possible this occurred through loss of a protective variant in L, or gain of a risk variant in M and N. Haplogroup N, which is enriched in populations that migrated into Europe, harbors variants 10398A and 8701A. The 10398A variant is associated with alterations in mitochondrial membrane potential and calcium metabolism [36]. The 10398A variant has lesser mitochondrial coupling efficiency and greater heat production than L haplogroups (10398G), and is postulated to be a key determinant in the adaptation of populations to the colder climates in northern Europe [8, 37, 38]. However the picture is complex, since 10398 appears to be a hypermutable site [39] and the 10398G variant is seen in J, K, L and M haplogroups, whereas U, T, H and V carry 10398A. This suggests that other mitochondrial variants and the mtDNA background play an important role.

In addition to their role in phylogenetics and adaptation, mtDNA mutations are linked to differences in metabolic rates and energy expenditure. The effects of mitochondrial haplogroups on mitochondrial function and bioenergetics as well as their association with different disease phenotypes and longevity have been studied clinically by evaluating whole body energy expenditure and oxygen consumption as well as their association with diseases and at the cellular and molecular levels using cybrid cell lines. Clinically, haplogroup L has been shown to have the lowest resting metabolic rate compared to the European haplogroups [40]. And among the European haplogroups, haplogroup J has the lowest maximum oxygen consumption with the most significant difference being between haplogroups J and H [41, 42]. Cellular differences in oxygen consumption and oxidative-phosphorylation capacity have been shown using cybrid cells with L haplogroup showing most efficient oxidative-phosphorylation capacity compared to the major European haplogroup H and among European haplogroups, J and UK haplogroups having the lowest coupling efficiency [9, 43–45]. The association of mitochondrial haplogroups with longevity and disease phenotypes seems to be due to the interdependence of mitochondrial energy dependence, reactive oxygen species production and mitochondrial activation of apoptosis [8, 38]. Tightly coupled haplogroups will use less calories to produce more ATP; however with increased calorie intake there will be increased ROS production predisposing to aging, neurodegenerative diseases and cancer [11, 12, 37, 46–50]. On the other hand uncoupled mitochondria generate less ATP per calorie consumed and are more prone for diseases related to energy insufficiency [44, 51–53]. With less ROS production, the uncoupled mitochondria are protected from aging, neurodegenerative diseases and cancer. Another example of disease state characterized by mitochondrial dysfunction is sepsis. Data are inconsistent on the association of European mitochondrial haplogroup with sepsis. Uncoupled mitochondria seem to be protective. In the study by Lorente et al, cluster JT was found to be associated with improved survival in sepsis in association with increased COX activity [54, 55]. Another explanation is the increased heat production and reduced ROS production with JT clade. In other studies, cluster HV and haplogroup H showed lower risk of severe sepsis compared to JT and J haplogroups and improved survival from sepsis compared to non H haplogroups [56, 57]. Unlike sepsis, PAH is characterized by cancer-like proliferation of pulmonary vascular cells and shift to glycolysis corresponding to energy deficient state. Oxidative phosphorylation uncoupling genomes associated with reduced aerobic production of ATP and production of ROS might be advantageous to the highly proliferative, apoptotis-resistant cells in the pulmonary vasculature and may explain the shift from oxidative phosphorylation to aerobic glycolysis.

Although the overall haplogroup distribution within our sample is consistent with the known distribution of haplogroups within Europeans and Africans (http://www.mitomap.org), the haplogroup distribution within African-descent individuals differed between PAH and healthy controls, with a significantly lower proportion of L haplogroups in patients than in the control group. Mitochondrial haplogroups with lower coupling efficiency increase predilection to energy deficiency diseases. For example, haplogroup J is associated with Leber hereditary optic neuropathy and both haplogroups J and K increase susceptibility to multiple sclerosis [41, 44, 51]. The increased risk of PAH with less coupled haplogroups is in agreement with this overall concept, since pulmonary vascular cells in PAH lesions are well described to have shift to glycolysis corresponding to energy deficient state [14, 15, 19]. The greater association of branches JT and UK with PAH as compared to L even after removal of familial cases and known PAH-associated mutations indicates that the mitochondrial genomic risk is independent of heritable PAH mutations. The lack of any reported signal for matrilineal inheritance patterns within PAH families likely reflects the very high relative risk conferred by a mutation in *BMPR2* or other heritable PAH genes, which dwarfs the odds ratios for mitochondrial haplogroups reported here. However, mitochondrial haplogroups could modify the penetrance of PAH mutations within families, and this warrants further study.

Haplogroup M was also associated with PAH risk, but in contrast to N, haplogroup M (10398G and 10400C variants) has tight coupling of oxidative phosphorylation, high levels of ATP production and minimal heat generation, suggesting there may be a different mechanism for PAH risk. It is important to note that this association is based on only 7 individuals (6 patients and 1 control), compared to more than 40 individuals each in the L, HV, JT and UK groups. The highest frequency of M occurs in Asia, however we excluded 9 Asian subjects with PAH (4 with the M haplogroup and 5 undetermined) because there were only 2 Asian individuals in our control group. Additionally, we excluded 13 individuals (4 controls and 9 patients) with Hispanic ethnicity for whom race was unknown, thus precluding case-control matching for race. Of these, 4 individuals (3 patients, 1 control) had the M haplogroup and 9 were undetermined. A larger study is therefore required to confirm the M-haplogroup association.

Mitochondrial haplogroups can also influence susceptibilities to, and severity of clinical diseases through mechanisms independent of bioenergetics. Differential susceptibilities to diseases found in some populations have been assigned to mtDNA through its influence on nuclear gene expression and consequently cellular functions [9]. This has been tested using cybrids, which are created by transfer of mitochondria with specific mtDNA haplogroups into human cell lines with identical nuclear DNA. For example, while L cybrids have lower mtDNA copy numbers and more efficient mitochondrial coupling as compared to H cybrids [9], the L and H cybrids also have differences in expression of nuclear genes. The L cybrids have lower expression levels of complement pathway and innate immunity genes and higher levels of inflammation-related signaling genes [9]. Thus, the vulnerability of specific haplogroup populations to PAH may occur through alterations of cellular pathways as well as through changes in bioenergetics.

The small number of African Americans with PAH in the study is a potential limitation. Prospective matching by race between PAH and control groups prior to genotyping would have been preferable. Nonetheless, with13 PAH and 33 control African American samples, the study has a power of 80% to detect a difference of 28% in the ratio of haplogroup L (L0/L1/L2 and L3) for controls, assuming that the ratio is 69% in PAH patients. In fact, the number of African Americans in this cohort is similar to numbers described in the multisite Registry to Evaluate Early And Long-term PAH Disease Management (REVEAL), a U.S. observational prospective registry that consecutively enrolled patients with group I PAH (NCT00370214) [58]. Although studies have not identified race as a risk factor to develop PAH, the REVEAL registry also contained primarily Caucasians (12% African Americans and 72.5% Caucasians), which is similar to the distribution in this cohort [58]. The study only has 46 African Americans (13 PAH and 33 controls), which is then underpowered for the comparison of PAH rates among different haplogroups. We then dichotomized the haplogroups into L (L0/L1/L2 and L3) and others. We found that L was represented among 97% African American controls, as compared to 69% among African American PAH individuals, showing significant protective effect of L. African-Americans have been primarily described to have pulmonary hypertension in association to left heart disease, lung diseases or sickle cell disease, as opposed to idiopathic PAH [59]. In support of a possible protective effect of African ancestry, anthropologic studies show that Ethiopians on the Simian plateau do not suffer from the pathologic elevated pulmonary vascular resistance characteristic of Andeans in response to long-term hypoxia at high altitudes despite very low oxygen saturations [60].

Overall, the findings suggest that mitochondrial DNA polymorphisms may influence risk of PAH. The haplogroup L is associated with a lower risk of PAH, whereas haplogroups M, and the N branches of HV, UK and JT may be associated with an increased risk for developing the disease. Further work is needed to determine the mtDNA variants, their interactions with nuclear genes, and their mechanisms of effects on pulmonary vascular cells that lead to susceptibility or resistance to PAH. However, the results suggest that a vulnerability to PAH may have emerged with migration of populations out of Africa to environments that selected for variants advantageous to life in the new climates but with tradeoff of risk for bioenergetic-related diseases.
